# Post-hemodialysis Retro-Odontoid Pseudotumor Without Atlantoaxial Instability or Rheumatoid Arthritis: A Case Report

**DOI:** 10.7759/cureus.80237

**Published:** 2025-03-07

**Authors:** Nayef M Aluzime, Abdulrahman Tabbaa, Abdulrahman Alarjani, Fayez AlTabbaa

**Affiliations:** 1 Spine Surgery Department, Prince Sultan Military Medical City, Riyadh, SAU; 2 Family and Community Medicine Department, King Saud University, King Saud University Medical City, Riyadh, SAU

**Keywords:** cervical myelopathy, corpectomy, meningitis, pseudotumor, retro-odontoid

## Abstract

Retro-odontoid pseudotumor is commonly associated with rheumatoid arthritis and C1-C2 instability. Meningitis has not been described as an associated factor for retro-odontoid mass before. Retro-odontoid mass as a pathology could be a serious condition. In this case report, the patient had a significant neurological deficit as he had a score of grade B on the American Spinal Injury Association Impairment Scale (ASIA). To our knowledge, approaching the lesion through the anterior approach with C3 corpectomy was not described before in the literature. We are reporting this case to increase the awareness of spine surgeons about the rarity of this case and the benefits of direct decompression over indirect decompression as per our experience.

## Introduction

A retro-odontoid pseudotumor is a non-neoplastic, fibroreactive tissue involving the odontoid process, which can cause cervical stenosis, cord compression, and myelopathy. The prevalence and incidence of retro-odontoid pseudotumors are unclear due to the rarity of reported cases.

The exact causes of retro-odontoid pseudotumors are not well-known. Different diseases are associated with retro-odontoid pseudotumors, such as rheumatoid arthritis and psoriatic arthritis [1,2]. Other less common associated conditions are unstable odontoid fractures, post-traumatic pseudoarthrosis of the odontoid process, os odontoideum, cervical instability post-laminoplasty kyphosis, hemodialysis, craniocervical junction malformations, and chronic atlantoaxial instability [3,4-7].

Different surgical approaches and procedures for managing retro-odontoid pseudotumor have been reported, including anterior transoral, posterior, far lateral approach, C1 laminectomy without fusion, occipitocervical fusion, and atlantoaxial fusion [8-10]. However, in literature review on retro-odontoid pseudotumors, a C3 corpectomy was not reported as an option for surgical management. In this report, direct decompression of the mass provides better results in the form of improvement of neurological findings in the patient compared to indirect decompression posteriorly.

## Case presentation

A 45-year-old man known to have hypertension, diabetes mellitus, and end-stage renal disease (ESRD) on regular hemodialysis three times per week presented to the emergency department with neck pain associated with right upper and lower limbs numbness and weakness. He has a history of unsteady gait and hand dexterity issues, which started three weeks before the presentation. The patient denies a history of fever, weight loss, or night sweats. He had been diagnosed with bacterial meningitis and treated with IV antibiotics at another hospital four months earlier.

**Figure 1:**
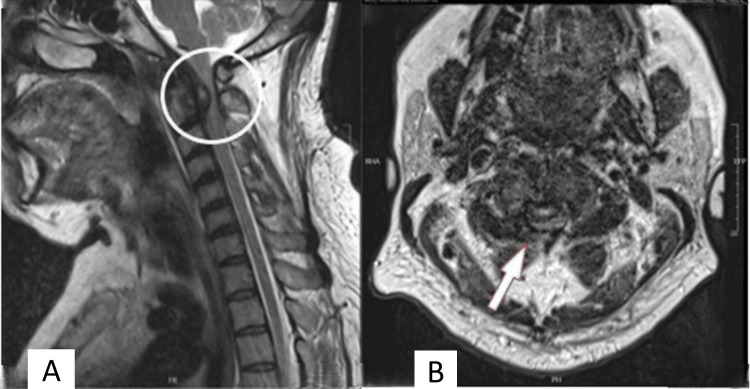
MRI sagittal (A) and axial (B) views of the cervical spine revealed significant cord compression at the level of C2 due to an oval-shaped mass behind the odontoid process (indicated by circle and arrow, respectively).

**Figure 2:**
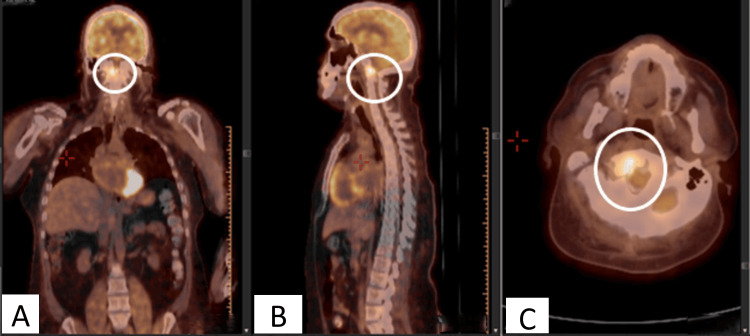
PET scan with coronal (A), sagittal (B), and axial views (C) showed the odontoid has high metabolic activity, suggesting infection (circles).

On presentation, he was afebrile. He had weak right-hand grip strength with power 3/5 and finger abduction 3/5, decreased sensation C6, C7, and C8 dermatomes with hyporeflexia, scoring grade D as per the American Spinal Injury Association Impairment Scale (ASIA). Laboratory investigation showed a white blood cell count (WBC) of 5.54 1000/μl, C-reactive protein (CRP) of 16.34 mg/L, erythrocytes sedimentation rate (ESR) of 31 mm/hr, negative blood culture, negative sputum culture, negative urine culture, and negative blood culture for brucella and tuberculosis (Table 1). Magnetic resonance imaging (MRI) of the cervical spine showed significant cord compression at the level of C2 due to an oval-shaped mass behind the odontoid process (Figure 1). A positron emission tomography (PET) scan showed that the odontoid had high metabolic activity, suggesting infection (Figure 2).

**Table 1:** Laboratory Data

Variable	On Admission	Reference
WBC	5.54 1000/μl	(4.5–11) 1000/μl
C-Reactive Protein	16.34 mg/L	<8 mg/L
Erythrocyte Sedimentation Rate	31 mm/hr	(0–13) mm/hr
Blood culture	negative	
Sputum culture	negative	
Urine culture	negative	
Blood culture for brucella	negative	

During the 1st two weeks of hospitalization, different specialty teams were consulted, including internal medicine, nephrology, physical therapy, and infectious disease. Different imaging, including MRI, PET scan, and laboratory investigations, were carried out. In the meantime, the patient’s weakness progressed to 0/5 muscle power over the right upper limb, left-hand grip 3/5, and left-hand finger abduction 3/5. He had also developed right lower limb weakness, hip flexion 3/5, knee flexion and extension 4/5, ankle dorsiflexion, and plantar flexion 3+/5 with decreased sensation all over the right side. He had negative upper motor neuron signs.

**Figure 3:**
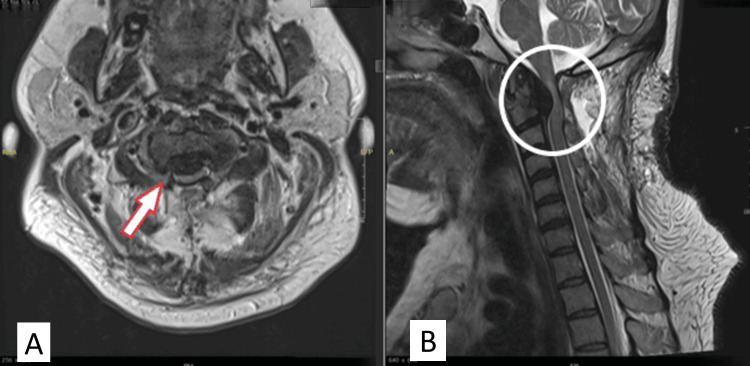
Post-operative cervical MRI. The axial view (A) showed significant cord compression by a retro-odontoid mass (arrow). The sagittal view (B) showed an area of posterior decompression with mild improvement of cord compression in comparison to the initial MRI and abnormal hypersignal intensity of the spinal cord (circle).

Posterior cervical decompression of C1-C2 by laminectomy with preservation of facet joints to avoid iatrogenic instability without fusion was done for the patient and a small biopsy was taken, which was negative for culture. The histopathology report was inconclusive. Post-operatively, the patient started on dexamethasone, which was tapered over three days. Right-hand weakness showed improvement, with muscle power of 2/5. The patient began an extensive course of physical therapy and a rehabilitation program. A post-operative MRI was done, and mild improvement was visible in cord compression (Figure 3).

**Figure 4:**
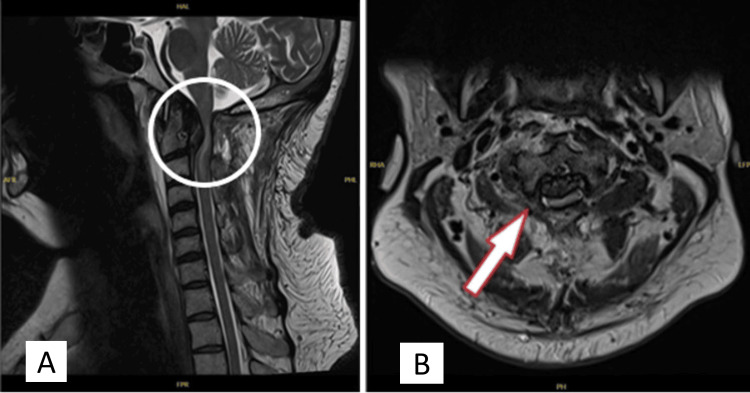
Follow-up (2 months post-decompression) MRI of the cervical spine with axial (A) and sagittal views (B) showed spinal cord compression and interval progression of the intramedullary signal hyperintensity of the cord (indicated by circle and arrow, respectively).

Two months post-operatively, the patient had a recurrence of right upper limb weakness with a global power of 0/5. He had a decrease in sensation, scoring grade B as per the ASIA impairment scale. He also had progressive weakness of the right lower limbs with a power of 2/5 and was not able to walk. Follow-up cervical MRI with contrast showed chronic cord compression at the C2 by an oval-shaped structure posterior to C2, which shows peripheral enhancement on post-contrast images (Figure 4). Again, compression was noted to be more severe to the right of midline with mild interval progression of the intramedullary hyperintensity at this level, most likely representing changes of myelomalacia.

**Figure 5:**
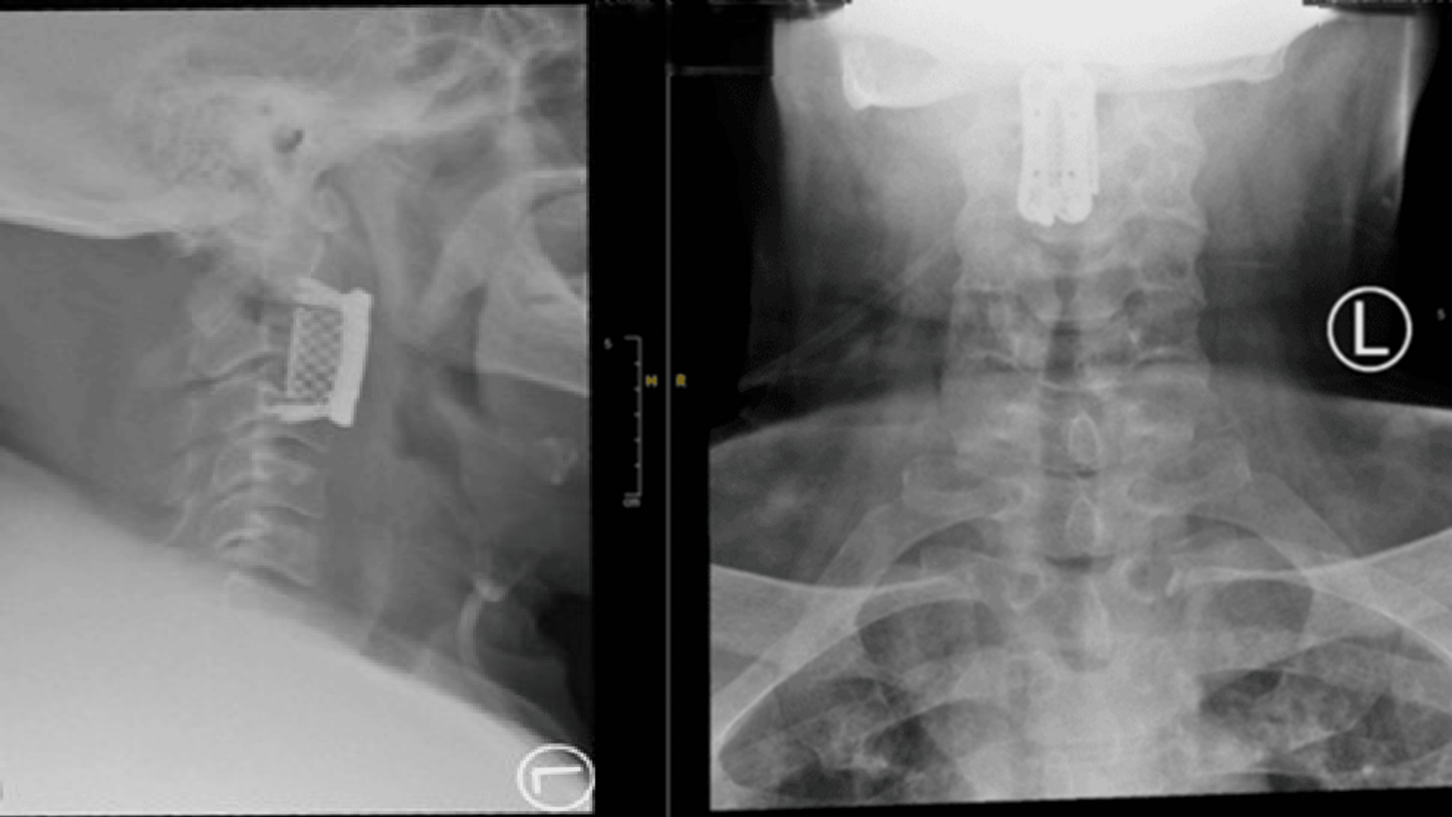
Post-operative radiograph showed a satisfactory position of the cage.

Many approaches for retro-odontoid masses were described in the literature. The decision to perform a C3 corpectomy was made to facilitate the excision of the retro-odontoid mass. The patient underwent a C3 corpectomy through a classic anterior cervical approach. The posterior corner of the C2 end plate was partially excised without affecting the central part of the end plate to allow for enough space to decompress the mass. A cheese-like pus was drained, and a sample was obtained for cultures and histopathology. Then, a mesh cage was applied and was fixed with a plate and screws from C2 to C4 (Figure 5). In the early post-operative course, the patient significantly improved in terms of muscle power. The results of soft tissue cultures and swabs were negative. The histopathology report showed fragments consistent with tumoral calcinosis.

**Figure 6:**
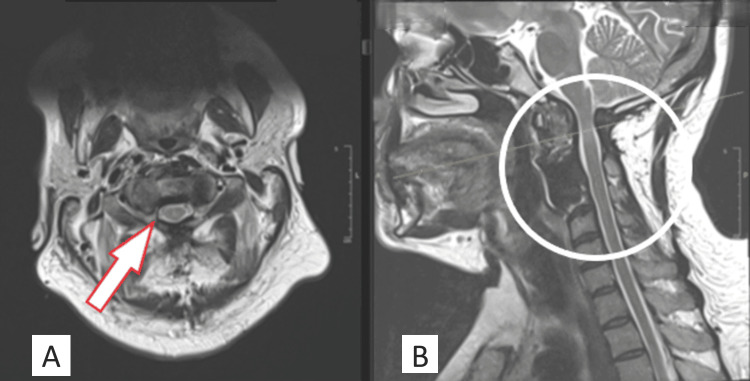
MRI done six months post-operative. The axial view (A) showed improvement of the cord compression with CSF all around the cord and full excision of the mass (arrow). The sagittal view (B) showed the hypodensity signal at the site of C3 corpectomy, with no sign of collection, adequate spinal cord decompression, and improvement of intramedullary signal hyperintensity of the spinal cord (circle). CSF: cerebrospinal fluid

The patient was seen six months post-operatively in the clinic, and upon assessment, he had improved hand dexterity, although he was still complaining of an unsteady gait. The muscle power was 5/5 in all lower and upper limbs muscles, with intact sensation, scoring grade A as per the ASIA scale. MRI at six months post-operatively was done and showed significant improvement in cord compression (Figure 6).

## Discussion

Retro-odontoid mass causing a significant compression over the spinal cord can be a serious condition that can lead to progressive neurological deficit and gait unsteadiness. Although hemodialysis has been identified as an associated factor, the patient's condition began after being diagnosed with meningitis [7]. This gives an insight into the true role of hemodialysis as a predisposing factor and raises a question about the role of meningitis as a predisposing factor.

Indirect decompression by posterior cervical decompression with or without fixation is an option for the management of retro-odontoid pseudotumor [9]. In our case, after C1-C2 posterior decompression without fixation, there was a temporary mild improvement of the patient’s weakness, followed by a significant progression of weakness, with an increase in the size of the retro-odontoid mass, as shown in an MRI (Figure 3).

Approaching the mass directly through C3 corpectomy with trimming the posterior part of lower end plate of of C2 without jeopardizing the integrity of end plate for cage buttrsing gave us enough space to completely excise the lesion directly, which would be difficult with other approaches as the mass was in the anterior part of the canal without side extensions. A complete mass excision leads to good patient outcomes in post-operative neurological assessment. 

## Conclusions

A retro-odontoid mass is a challenging case. Its proper management depends on various factors, including the patient's presentation and the surgeon's preferred approach. Providing enough decompression can play an important role in preventing recurrence*.* Different approaches have been reported for the surgical management of retro-odontoid masses. C3 corpectomy can be used as an option to provide access to excise retro-odontoid masses and to provide adequate cord decompression.
